# Analyzing the effect of neoadjuvant stereotactic ablative body radiotherapy on pancreatic tumor perfusion using computed tomography perfusion

**DOI:** 10.3389/fonc.2026.1677923

**Published:** 2026-03-12

**Authors:** Jin-Young Bang, Dan Breadner, Timothy Nguyen, Anton Skaro, Chris Goodman, Stephen Welch, Elena Tsvetkova, John Lenehan, Ken Leslie, Ephraim Tang, Douglas Quan, Michael Sey, Brian Yan, Danielle Porplycia, James Sinfield, Stewart Gaede, Ting-Yim Lee

**Affiliations:** 1 Department of Medical Biophysics, Western University, London, ON, Canada; 2 London Health Sciences Centre, London, ON, Canada; 3 Robarts Research Institute, London, ON, Canada; 4 Department of Oncology, Western University, London, ON, Canada; 5 Department of Surgery, Western University, London, ON, Canada; 6 Department of Medicine, Western University, London, ON, Canada; 7 Department of Oncology, London Health Sciences Centre, London, ON, Canada; 8 Lawson Research Institute, London, ON, Canada

**Keywords:** computed tomography perfusion (CTP), extravascular extracellular space, neoadjuvant therapy, pancreatic ductal adenocarcinoma PDAC, stereotactic ablative body radiotherapy (SABR), tumor perfusion

## Abstract

**Purpose:**

The objective of this study was to investigate the feasibility of administering neoadjuvant stereotactic ablative body radiotherapy (SABR) for the purpose of increasing pancreatic tumor perfusion, hence potentially improving systemic drug delivery in patients with resectable (RPC) or borderline-resectable pancreatic cancer (BRPC).

**Methods:**

Neoadjuvant SABR was administered to RPC (n=1) and BRPC (n=5) patients with a dose prescription of 27–30 Gy in 3 fractions and a dose boost up to 45 Gy to the metabolically active areas of the tumor. Computed tomography perfusion (CTP) studies were acquired at baseline, 6 hours after delivering the first SABR fraction (post-1^st^-fx), and 3–4 weeks after completing SABR (post-RT). For BRPC patients, studies were also acquired after neoadjuvant chemotherapy (post-cx), which preceded SABR. Using a deconvolution-based CTP software, the tumor blood flow (BF), blood volume (BV), permeability-surface area product (PS), and extravascular extracellular volume (V_e_) were calculated at each study instance. Additionally, a surrogate measure of the tumor cell density (CD) was derived from V_e_.

**Results:**

The post-1^st^-fx tumor BF was significantly higher than baseline (p=0.010) for the RPC patient and 2 other BRPC patients who had acquired a pre-treatment baseline scan. The post-RT BF, PS, V_e_ were also significantly higher than baseline (p<0.05), and a decrease in CD could be observed after the initial treatment (% decrease relative to baseline: post-cx for BRPC = 23%; post-RT for RPC = 14%). The BRPC patients showed a subsequent decrease in CD at post-RT relative to post-cx (<5%). For all BRPC patients, the post-1^st^-fx BF was significantly higher relative to post-cx (p=0.033), followed by a non-significant decline at post-RT. Conversely, the PS showed a significant decrease at post-1^st^-fx relative to post-cx (p<0.001), although the post-RT changes showed no consistent trend. The V_e_ was still significantly higher at post-RT relative to post-cx (p=0.015).

**Conclusion:**

This study provides preliminary evidence that delivering SABR in the neoadjuvant setting for RPC and BRPC patients can induce acute and sustained increases in pancreatic tumor perfusion. Additionally, this study shows that tumor CD may have adequate sensitivity as a biomarker for monitoring patient response to standard-of-care therapy.

## 1 Introduction

Pancreatic cancer has the lowest 5-year relative survival rate (13%) of all the cancer types in the United States (1). While routine surveillance for the more susceptible population has enabled diagnosis and surgical intervention at an earlier stage, overall survival is still very poor as the majority of patients present with advanced-stage pancreatic cancer at diagnosis (2, 3). Currently, surgical resection is the primary curative option for pancreatic cancer. However, surgical intervention is only feasible for patients with resectable pancreatic cancer (RPC), which is non-metastatic and well-localized without any involvement of the surrounding critical vessel structures, including the superior mesenteric artery and vein, celiac axis, common hepatic artery, and the portal vein. Some cases with non-negligible vascular involvement may still be marginally amenable to resection with vessel reconstruction—known as the borderline-resectable pancreatic cancer (BRPC) patients (4–6). Successful surgical removal of the tumor followed by adjuvant therapy has demonstrated that it could increase the 5-year survival (5YS) of patients up to approximately 20% (7). Retrospective studies investigating the resection outcomes of pancreatic cancer patients have also reported that the actual 5YS of patients who had no nodal metastases and received negative-margin resection (R0) was 38.2%, and those with exclusively favorable conditions for resection had a much higher 5YS of 50%, which strongly suggests that an R0 resection could significantly improve patient survival outcome (8). To maximize the chances of achieving an R0 resection, neoadjuvant therapy (NAT), such as neoadjuvant chemotherapy, is often implemented in treatment regimens as it can help downstage the tumor conditions by reducing its size, minimizing vascular involvement, and preventing possible metastasis prior to resection (9, 10).

Approximately 90% of all pancreatic cancer patients are diagnosed with pancreatic ductal adenocarcinoma (PDAC), or malignancies derived from the ductal cells lining the epithelium of the pancreatic duct (2). PDAC tends to spread rapidly to the lymphatic system and other organs, and this highly metastatic predisposition contributes significantly to the low survival rate of pancreatic cancer patients (11, 12). Additionally, chemotherapeutic drugs that are commonly used in systemic NAT are often less effective in treating PDAC patients due to the presence of a dense, fibrotic tumor microenvironment, also known as the stroma (13, 14). Fibroblasts and pancreatic stellate cells (PSCs) largely contribute to the cellular components of the tumor stroma, along with other immune cells such as the tumor-associated macrophages (13, 14). Constituents of the extracellular matrix (ECM), including polysaccharides like hyaluronan; and proteins, such as laminin, fibronectin, and collagen types I, III, and IV comprise the non-cellular stromal components (13). In PDAC tumorigenesis, the normally quiescent PSCs become activated and upregulate the expression of alpha-smooth muscle actin (15–17). The rapid proliferation of cancer-associated fibroblasts (CAFs) and activated PSCs as well as the increased secretion of ECM components by the stromal cells define the hallmarks of desmoplasia, a fibrotic reaction by which PDAC develops its characteristic stroma with high tissue density and elevated interstitial fluid pressure (IFP) (13, 18, 19). Consequently, pancreatic tumor perfusion is significantly reduced in comparison to the normal, healthy pancreatic tissue (19–22). Therefore, the desmoplastic stroma introduces a significant barrier to effective treatment for PDAC as its highly limited tumor perfusion hinders the systemic delivery and distribution of cancer drugs to the tumor volume, thus leading to chemoresistance and compromised efficacy of systemic drugs in NAT (14, 23–25).

Interestingly, recent studies in rectal and liver cancer have demonstrated that hypofractionated radiotherapy could produce acute vascular effects that increase tumor perfusion shortly after treatment (26, 27). While chemoradiotherapy (CRT) is already the current standard of care for the unresectable, locally advanced pancreatic cancer patients, the typical dose fractionation scheme in CRT delivers approximately 2–3 Gy per fraction (28), which is lower than the proposed radiation dose necessary to induce an increase in tumor perfusion (29). In contrast, higher doses of 9–10 Gy per fraction or greater can be delivered using stereotactic ablative body radiotherapy (SABR), which can precisely deliver high doses of radiation for tumor ablation in only 3–5 fractions with the guidance of an integrated imaging system (30). However, there remains a lack of clinical studies in the literature investigating the effect of high-dose irradiation specifically on pancreatic tumor perfusion. Thus, the objective of this study was to analyze the effect of neoadjuvant SABR on pancreatic tumor perfusion, specifically in patients with RPC and BRPC, by monitoring changes in tumor perfusion over the course of their treatment using computed tomography perfusion (CTP). It was hypothesized that neoadjuvant SABR could induce an increase in pancreatic tumor perfusion, which may potentially enhance the delivery of systemic NAT to the tumor site and help overcome PDAC chemoresistance.

## 2 Methods

### 2.1 Patient accrual

Six patients aged 18 or older with RPC (n = 1) or BRPC (n = 5) were prospectively recruited with informed consent, provided that they had histologically confirmed primary pancreatic cancer and were medically fit to undergo surgical resection. The BRPC patients received neoadjuvant chemotherapy—either FOLFIRINOX (2-week chemo cycles) or gemcitabine with Abraxane^®^ (4-week chemo cycles)—prior to undergoing neoadjuvant SABR. In contrast, the RPC patient only received neoadjuvant SABR with no accompanying NAT. Other inclusion criteria for the study included no evidence of distant metastases, life expectancy greater than 6 months, and adequate renal function (eGFR > 35 mL/min) to eliminate from the body the contrast agent used in CTP. Patients with severe comorbidities, recurrent pancreatic cancer, prior abdominal radiation, and disease involving the duodenum or stomach were excluded from the study. RPC patients receiving NAT other than neoadjuvant SABR were also excluded.

### 2.2 Radiotherapy planning and delivery

Endoscopic ultrasound-guided placement of fiducial markers for tumor localization and image verification was required in all accrued patients prior to baseline imaging. Patients also underwent a CT simulation protocol consisting of a breath hold fast-helical CT (BH-CT) scan and a 4D-CT scan on the Canon Aquilion Exceed LB CT scanner (Canon Medical Systems, Otawara, Japan) for tumor motion assessment.

**Figure 1:**
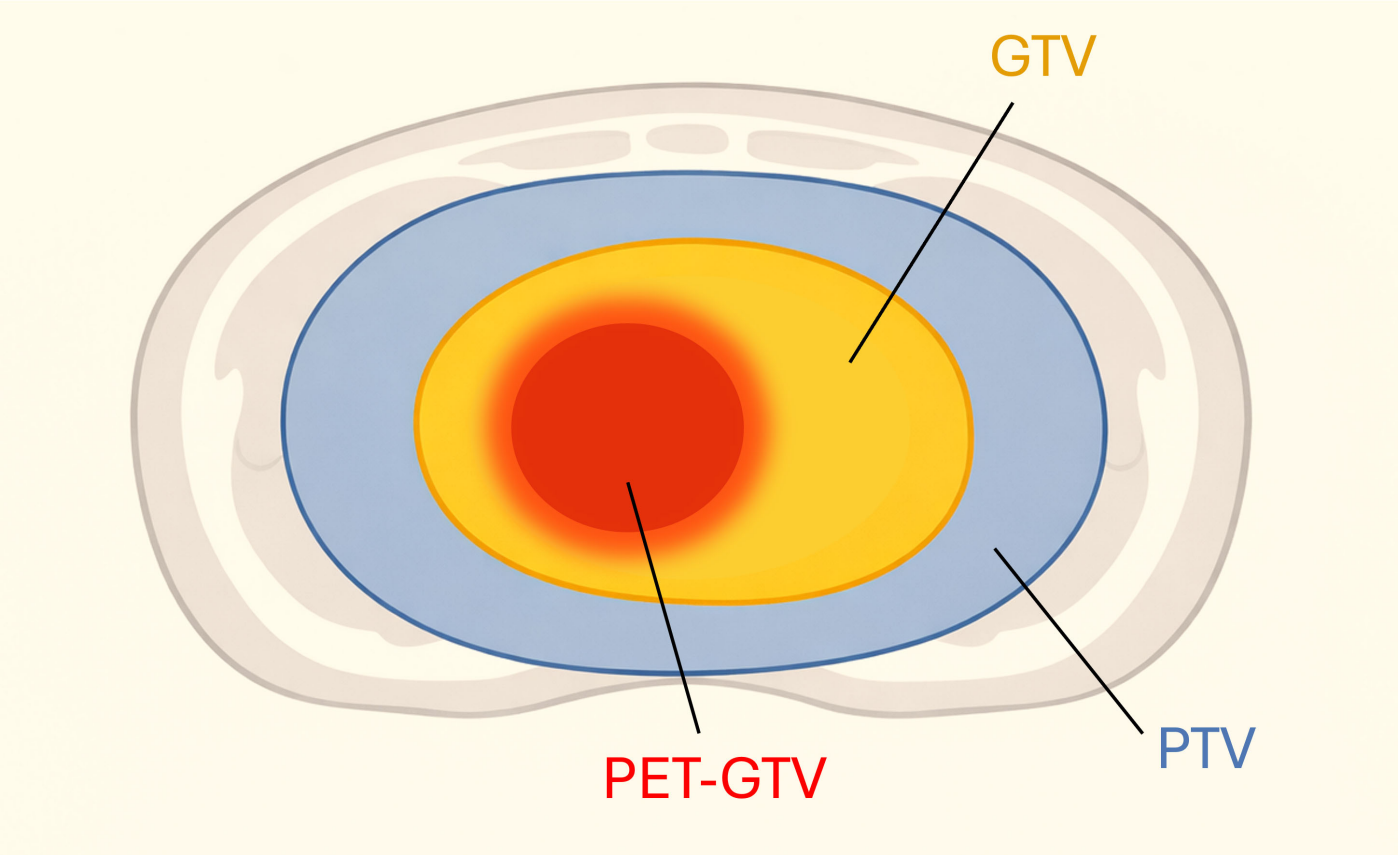
The planning target volume (PTV) includes a 5-mm margin added to the combination of the gross tumor volume (GTV) and the tumor vessel interference volume (TVI not depicted on figure). The metabolically active GTV (PET-GTV) represents a sub-volume within the defined GTV that has a standard uptake value (SUV) greater than 50% of the maximum SUV value on the acquired PET-GTV scan. The PET-GTV, GTV, and the PTV are illustrated in red, orange, and blue, respectively.

Computed tomography perfusion (CTP) and Hybrid PET/MRI using fluorodeoxyglucose (FDG) were also acquired from each patient prior to CT simulation. The gross tumor volume (GTV) was delineated by a radiologist using all available imaging information, including the planning CT, PET-MRI, and CTP. For radiotherapy, the planning target volume (PTV) was defined as a 5-mm volume margin added to the combination of the GTV and the tumor vessel interference (TVI) volume, or the circumferential segments of major vessels within 5 mm of the GTV. Metabolically active GTV (PET-GTV) was defined as the portion of the GTV with a standard-uptake value (SUV) greater than 50% of the maximum SUV on the acquired FDG-PET scan. A simple schematic illustrating the differences between the GTV, PET-GTV, and PTV are shown in **Figure 1**. The stomach, small intestine, duodenum, and large bowel were delineated on the planning CT, and a 3-mm margin was placed around each organ-at-risk (OAR) to define the planning-at-risk volumes (PRVs).

The SABR dose prescribed to the PTV ranged between 27–30 Gy in 3 fractions with an increased dose boost to the PET-GTV as high as dose constraints allowed, up to a maximum of 45 Gy. A PTV_eval structure, defined as the PTV minus the OAR PRV structures, was generated to ensure dose to 90% of its volume (D90%) was equal to at least 90% of the prescription dose or greater. In cases where the PTV coverage could not be achieved without exceeding the dose limits to the OARs, the PTV coverage or prescription dose was compromised, with a minimum prescribed dose of 21 Gy. All patients were treated using volumetric modulated arc therapy (VMAT) on a TrueBeam linear accelerator (Varian Medical Systems, Palo Alto, USA) with a built-in system for surface-guided radiotherapy (AlignRT, VisionRT, London, UK). All patients received deep inspiration breath-hold (DIBH) radiotherapy, except for one patient who needed end-expiratory gating. Each treatment session was set up to ensure reproducible positioning of the patient by performing direct tumor localization using cone-beam CT (CBCT) prior to treatment. For patients who received DIBH radiotherapy, the CBCT was acquired under DIBH conditions as well. In cases where the tumor was not visible on the CBCT, the implanted fiducial markers were used as surrogates for tumor position.

### 2.3 CTP imaging protocol

CTP images of the abdominal region encompassing the pancreatic tumor were acquired with an axial field of view of 16 cm (32 x 5 mm or 64 x 2.5 mm slices) using the GE Healthcare Revolution CT scanner (GE HealthCare, Waukesha, USA) at 120 kVp and 50 mAs. Image time intervals of 2.5 s and 15 s were used during the vascular (40–60 s) and interstitial phase (100–120 s), respectively, for a total acquisition duration of 3 minutes. An iodinated contrast agent was administered at a flow rate of 4 ml/s and dosage of 0.7 ml/kg via an antecubital vein after a delay of 5 s from the start of the scan. CTP images were acquired from both RPC and BRPC patients at multiple timepoints over the course of their treatment: a) at baseline; b) 6 hours after administering the first fraction of neoadjuvant SABR; and c) 3–4 weeks after completing SABR, just prior to surgery. For the BRPC patients, images were also taken after neoadjuvant chemotherapy, which preceded SABR.

### 2.4 Image and data analysis

**Figure 2:**
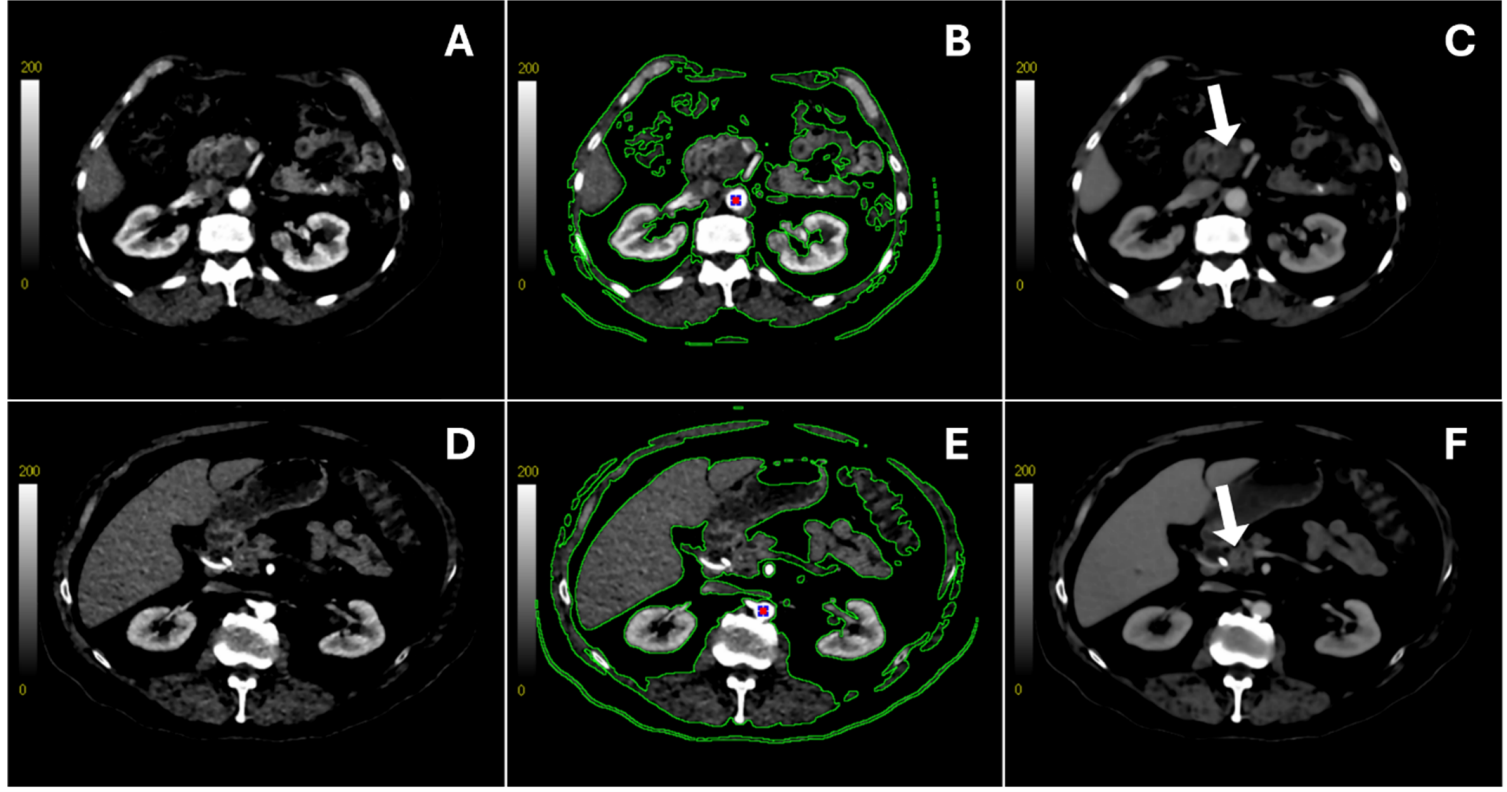
The top and bottom rows show the contrast-enhanced CT scan of one of the axial slices obtained from a borderline resectable patient (BRPC, Patient 2) and the resectable patient (RPC), respectively. Scans in the left column were acquired shortly after contrast injection **(A, D)**. The same scans are also shown in the middle column, with the segmented region enclosed in green showing where the deconvolution algorithm was applied to generate the perfusion maps **(B, E)**. The red dot shows the 3 x 3 pixel region of interest from which the arterial time-density curve (TDC) was obtained. The right column shows the average CT scan of the same axial slice **(C, F)**. The white arrow is pointing at the gross tumor volume (GTV).

A prototype deconvolution-based (31) CTP software (GE HealthCare, Waukesha, USA) was used to analyze the CTP scans. For each 32- or 64-slice volume of images acquired at different study instances, images were non-rigidly registered to minimize intra-study motion artifacts and align the anatomy between study instances for each patient. An average CT map was computed by calculating the average HU value across all timepoints at each pixel location. As shown in **Figure 2**, this average map was then segmented to include only the abdominal region of interest while excluding the background and other irrelevant structures. A 3 x 3 pixel region of interest (ROI) was placed at the center of the aorta to obtain the arterial time-density curve (TDC) from one of the axial slices. The deconvolution algorithm was then applied to the segmented average CT map to generate color-coded parametric maps for the following hemodynamic variables: blood flow (BF) (in mL/min/100 g of tissue), blood volume (BV) (in mL/100 g of tissue), and the permeability-surface area product (PS) (in mL/min/100 g of tissue). Using the delineated GTV contours as reference, 3 circular ROIs were drawn on different axial slices of the tumor volume on the segmented average CT map, which was also linked to its corresponding deconvolution-generated perfusion maps. When placing the ROIs, care was taken to avoid including the surrounding blood vessels, healthy pancreatic tissue, or the implanted fiducial markers. Mean values for BF, BV, and PS were calculated by taking the average of the enclosed pixel values within the ROIs.

The extravascular extracellular volume (V_e_) (in mL/100 g of tissue) was calculated by single-curve deconvolution, where an average tissue TDC was obtained from each of the 3 tumor ROIs and deconvolved with the arterial TDC. The calculated values from the 3 different ROIs were then averaged to obtain the final value for V_e_. The tumor cell density (CD) (in mL/g) was then calculated using the below equation (full derivation disclosed in the **Supplementary Material**):


$$CD=1-\frac{V_{e}}{100}$$

For statistical analysis, the patients were divided into 3 groups as depicted in **Table 1**. The Shapiro-Wilk test was used to test for sample data normality, and the one-tailed Student’s t-test was used to analyze the statistical significance (defined as p< 0.05) of changes in perfusion parameters between different study timepoints.

**Table 1:** Results from the one-tailed t-test comparing the tumor blood flow (BF), blood volume (BV), permeability surface area product (PS), and the extravascular extracellular volume (V_e_) between different timepoints.

*Group 1) RPC Patient + BRPC Patients 1 and 2.*			
Variable	p-value		
	Baseline to Post-1^st^-fx	Baseline to Post-RT	Post-1^st^-fx to Post-RT
BF	0.010*	0.020*	0.350
BV	0.149	0.082	0.344
PS	0.095	0.026*	0.145
V_e_	0.142	0.027*	0.072

Group 2) BRPC Patients 1 and 2.			
Variable	p-value		
	Baseline to Post-Cx	Baseline to Post-1^st^-fx	Baseline to Post-RT
BF	0.039*	0.039*	0.083
BV	0.035*	0.045*	0.094
PS	0.019*	0.043*	0.111
V_e_	0.058	0.009*	0.054

Group 3) All 5 BRPC patients.			
Variable	p-value		
	Post-Cx to Post-1^st^-fx	Post-Cx to Post-RT ^†^	Post-1^st^-fx to Post-RT ^†^
BF	0.033*	0.219	0.135
BV	0.173	0.092	0.078
PS	0.001*	0.082	0.355
V_e_	0.376	0.015*	0.061

* p-value< 0.05. The bolded values indicate statistically significant p-values less than a = 0.05.

† Did not include BRPC patient 3.

The patients were divided into 3 different groups based on the correspondence of the acquired set of CTP scans.

## 3 Results

**Figure 3:**
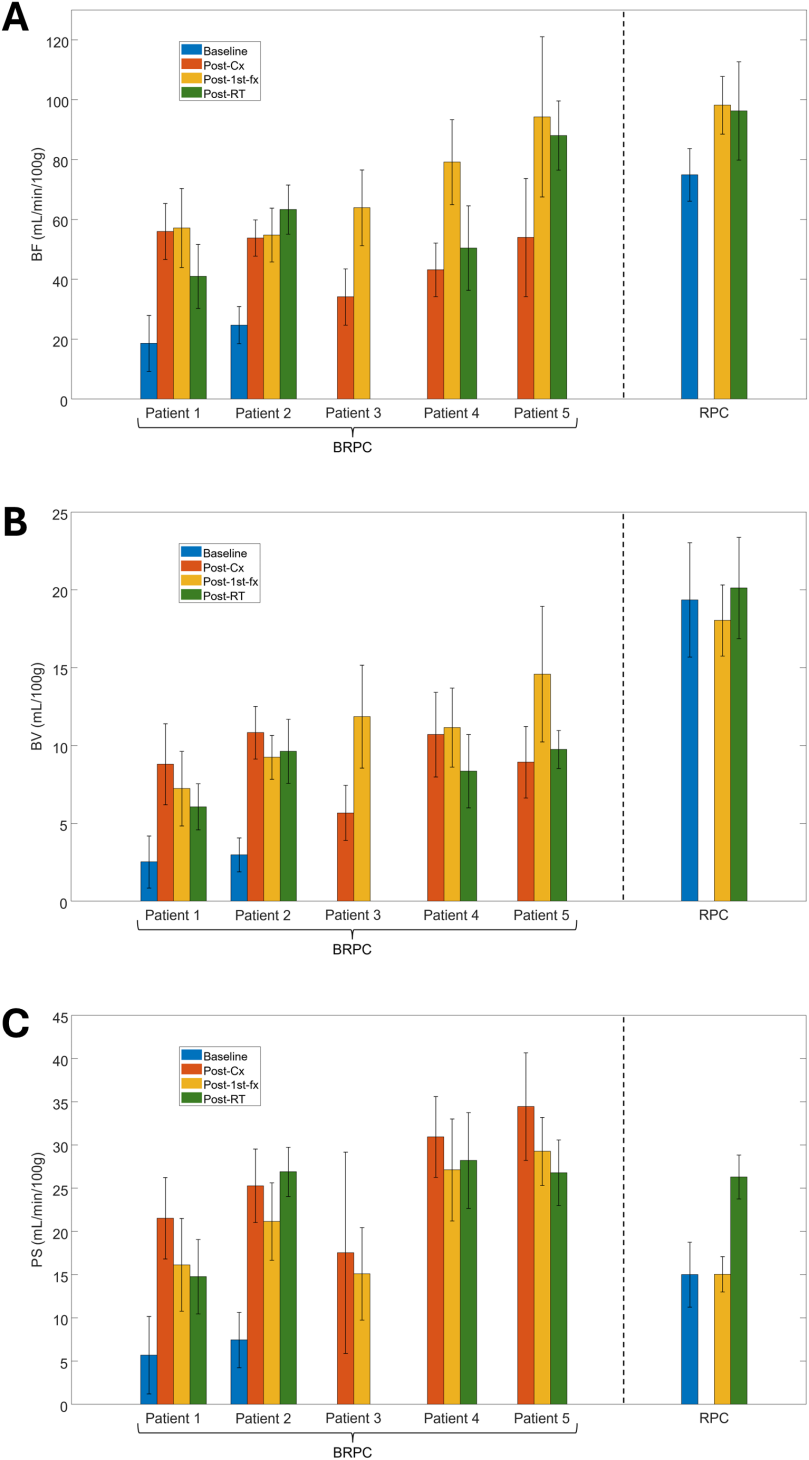
Grouped bar graphs showing the mean values of **(A)** blood flow (BF); **(B)** blood volume (BV); and **(C)** permeability-surface area product (PS) obtained from the 3 regions of interest (ROIs) placed within the gross tumor volume (GTV) on the deconvolution-generated perfusion maps. Mean values for each of the 5 borderline resectable patients (BRPC) and the resectable patient (RPC) are shown with error bars extending 1 standard deviation above and below the mean.

**Figure 4:**
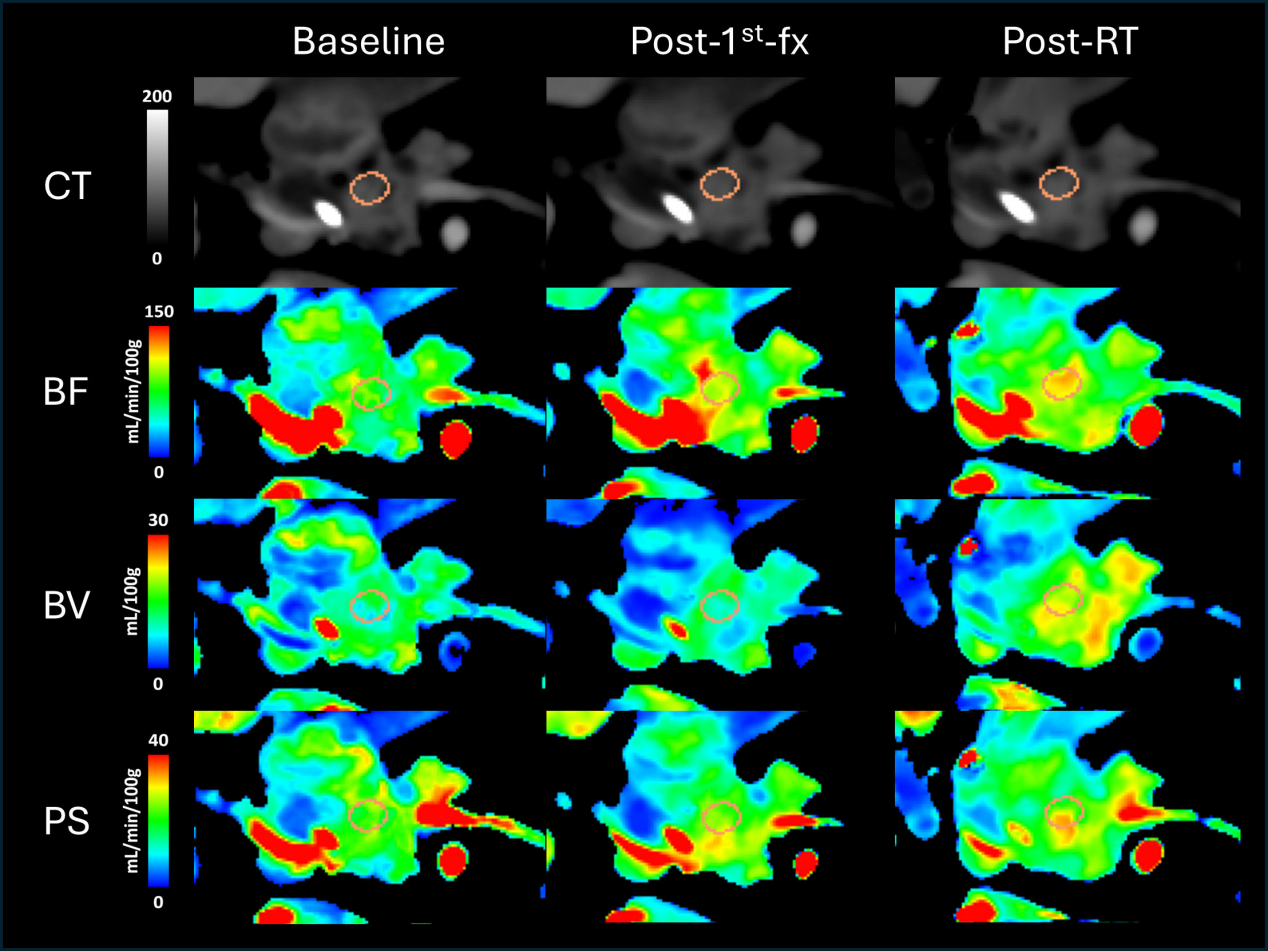
The top row shows the average CT scans of the resectable patient (RPC) showing 1 of the 3 circular regions of interest (ROIs) placed within the gross tumor volume (GTV), which is shown in orange. The rows below illustrate the corresponding color-coded perfusion maps showing blood flow (BF), blood volume (BV), and permeability-surface area product (PS). As shown in the color scale, red regions denote higher values, while blue regions denote lower values. Going from left to right, the 3 columns depict the different study instances at which the scans were acquired: pre-treatment (Baseline), 6 hours after delivering the first fraction of the 3-fx neoadjuvant SABR (Post-1^st^-fx), and 3–4 weeks after completing SABR (Post-RT), respectively.

**Figure 5:**
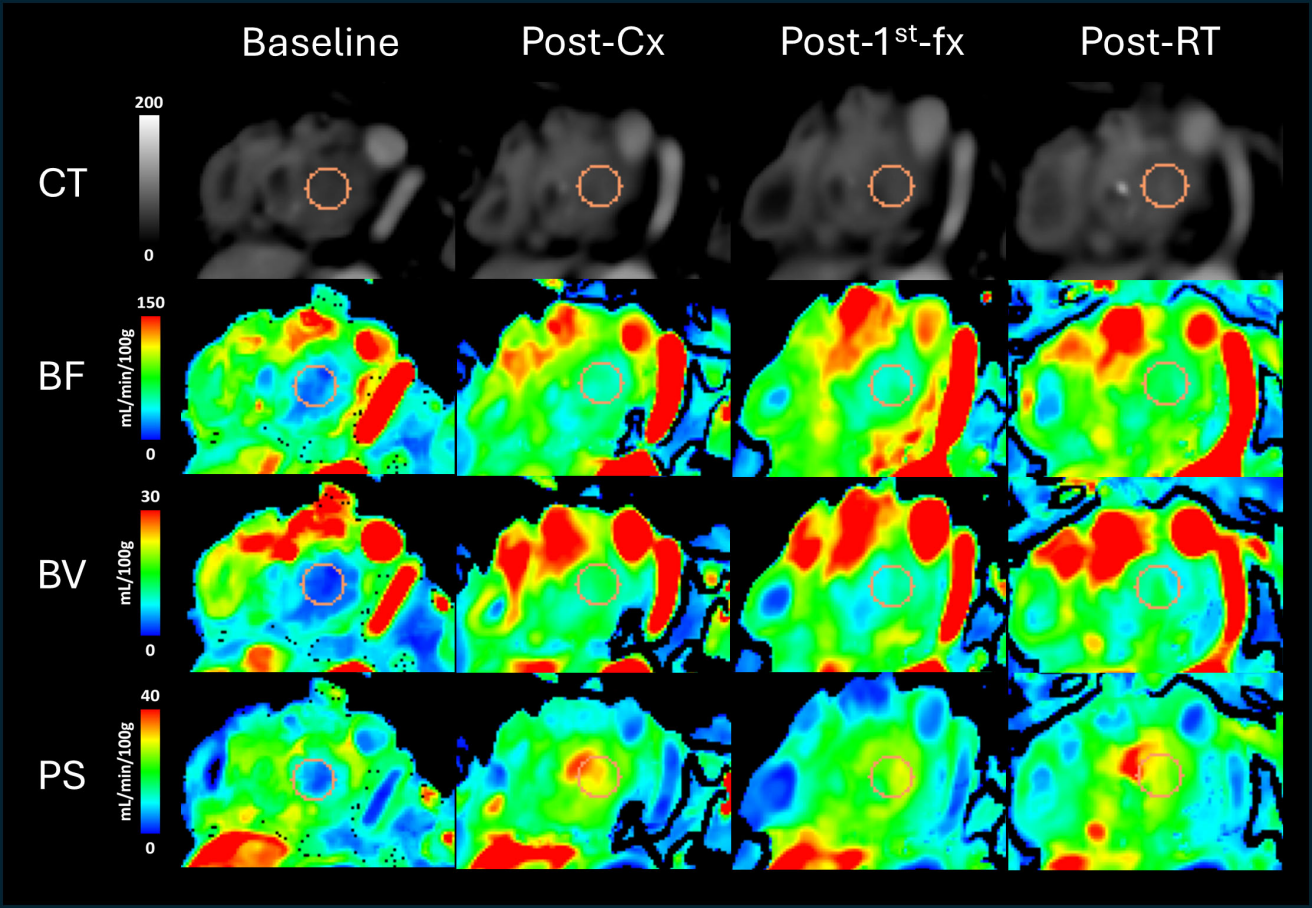
The top row shows the average CT scans of a borderline resectable patient (BRPC, Patient 2) showing 1 of the 3 circular regions of interest (ROIs) placed within the gross tumor volume (GTV), which is shown in orange. The rows below illustrate the corresponding color-coded perfusion maps showing blood flow (BF), blood volume (BV), and permeability-surface area product (PS). As shown in the color scale, red regions denote higher values, while blue regions denote lower values. Going from left to right, the 4 columns depict the different study instances at which the scans were acquired: pre-treatment (Baseline), after completing chemotherapy (Post-Cx), 6 hours after delivering the first fraction of the 3-fx neoadjuvant SABR (Post-1^st^-fx), and 3–4 weeks after completing SABR (Post-RT), respectively.

All CTP scans were successfully completed for the RPC patient as planned in the protocol. Out of the 5 accrued BRPC patients, only 2 patients (numbered as BRPC Patients 1 and 2) were able to acquire a baseline scan prior to receiving neoadjuvant chemotherapy. The other BRPC patients (BRPC Patients 3-5) had their first CTP scan acquired after completing chemotherapy as they had already started undergoing treatment at the time of accrual. BRPC Patient 3 was also unable to acquire the CTP scan at 3–4 weeks after completing SABR due to receiving early surgical intervention. Thus, a total of 19 CTP scans from 6 patients were analyzed in this study. The changes in the mean tumor BF, BV, and PS for all patients are shown in **Figure 3**. Qualitatively, the perfusion maps for the RPC patient and a sample BRPC patient (Patient 2) are illustrated in **Figures 4**, **5**, respectively.

For the RPC patient, the mean tumor BF at 6 hrs following the administration of the first fraction of neoadjuvant SABR (post-1^st^-fx) was approximately 30% higher relative to baseline. At 3–4 weeks after completing SABR (post-RT), there was little to no change in the mean BF relative to post-1^st^-fx (mean BF: baseline = 74.9 mL/min/100g, post-1^st^-fx = 98.2 mL/min/100g, post-RT = 96.3 mL/min/100g). Unlike the observed trend in BF, the mean PS did not change relative to baseline at post-1^st^-fx, but it showed an increase at post-RT (mean PS: baseline = 15.0 mL/min/100g, post-1^st^-fx = 15.0 mL/min/100g, post-RT = 26.3 mL/min/100g). There was also little to no net change in the mean BV across the study timepoints (mean BV: baseline = 19.4 mL/100g, post-1^st^-fx = 18.0 mL/100g, post-RT = 20.1 mL/100g). However, there was an overall increase in V_e_ at post-RT relative to baseline (mean V_e_: baseline = 20.98 mL/100g, post-1^st^-fx = 17.12 mL/100g, post-RT = 31.87 mL/100g).

As shown in **Table 1**, the RPC patient was analyzed in Group 1 with BRPC Patients 1 and 2, who also had a pre-treatment baseline scan for comparison. Notably, the tumor BF observed post-1^st^-fx was significantly higher than the pre-treatment baseline (p = 0.010). The post-RT BF, PS, and V_e_ were also significantly higher relative to baseline. However, BV did not show any significant trends in Group 1.

In Group 2, only BRPC Patients 1 and 2 were analyzed. Relative to baseline, all perfusion parameters were found to be significantly higher at post-1^st^-fx and post-cx, except for V_e_—which was close to reaching significance at post-cx (p = 0.058). However, no changes observed post-RT relative to baseline were found to be significant.

In Group 3, which consisted of all 5 BRPC patients, the post-1^st^-fx BF was found to be significantly higher than post-cx (p = 0.033). In terms of the post-RT response, except for Patient 2 who showed a continued increase in tumor BF; Patients 1, 4, and 5 showed a decrease in the mean tumor BF relative to post-1^st^-fx, which contributed to an overall decline in BF at post-RT. However, this was not found to be statistically significant. The mean PS showed a consistent decrease at post-1^st^-fx relative to post-cx (p< 0.001), although the post-RT change in PS was inconsistent and varied among patients. Interestingly, similar to what was observed in Group 1, BV did not show any notable trends while V_e_ was significantly higher at post-RT relative to post-cx (p = 0.015).

**Figure 6:**
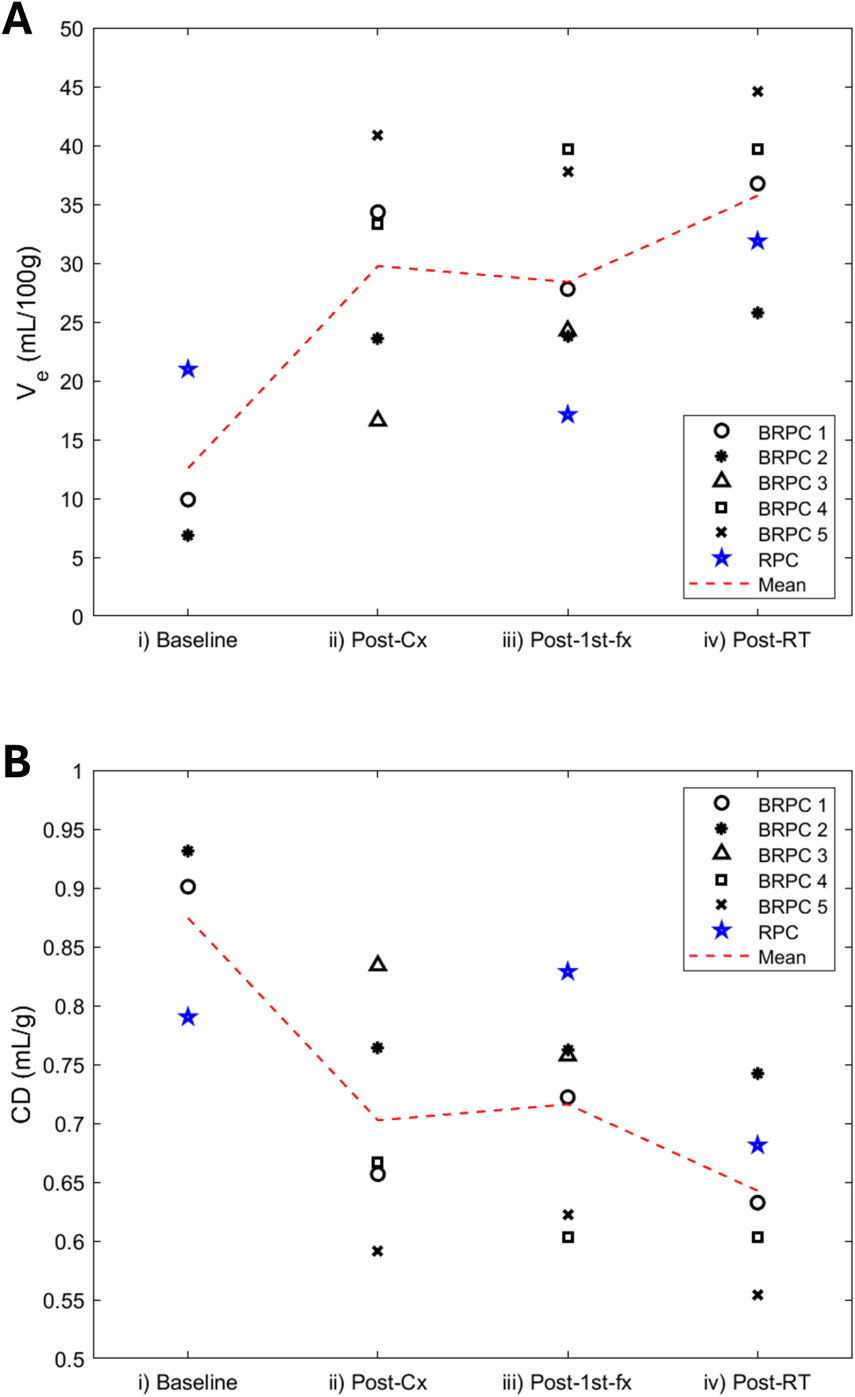
Scatter plot showing the change in **(A)** the extravascular extracellular volume (V_e_) and **(B)** the cell density (CD) over their respective treatment regimen for all 5 borderline resectable patients (BRPC) and the resectable patient (RPC). The mean value across patients at each study instance is illustrated with a red dotted line to show the overall change across the treatment timeline.

Lastly, as shown in **Figure 6**, there was an overall decline in CD over the course of treatment (i.e. overall increase in V_e_). The largest drops in CD occurred post-cx for Patients 1 and 2 in the BRPC arm (% decrease in CD relative to baseline = 23%) and post-RT for the RPC patient (14%). Subsequent drops (< 5%) could also be consistently observed between post-cx and post-RT for the BRPC patients. However, the acute changes in CD observed post-1^st^-fx varied among patients and did not seem to show a consistent trend as a group.

**Figure 7:**
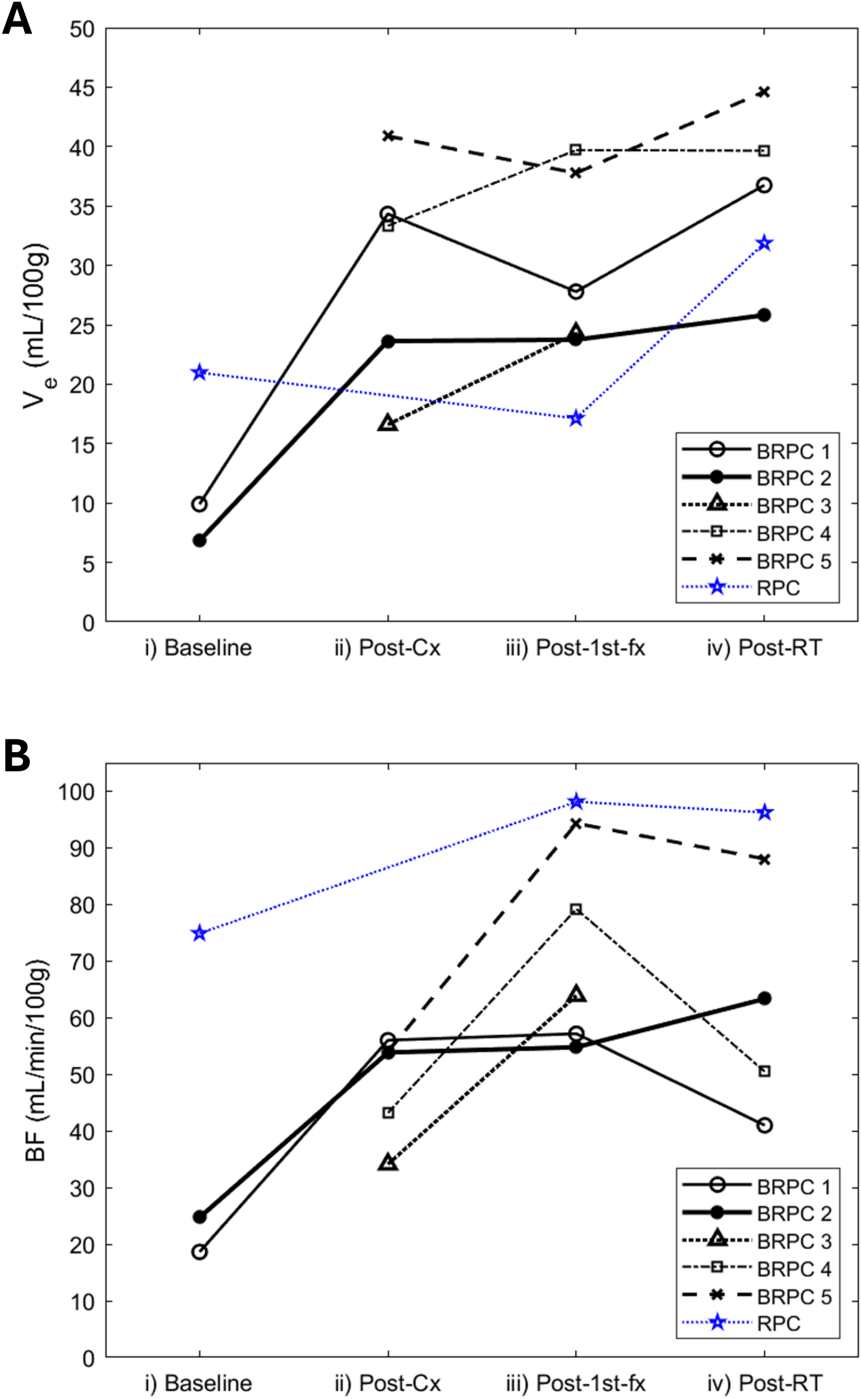
Line plot showing the change in **(A)** the extravascular extracellular volume (Ve) and **(B)** the blood flow (BF) over their respective treatment regimen for all 5 borderline resectable patients (BRPC) and the resectable patient (RPC). The individual patient trajectories are illustrated in dotted lines as defined in the plot legend.

For simplicity, individual patient trajectories for BF and V_e_ are illustrated separately in **Figure 7**.

## 4 Discussion

To the best of our knowledge, this is the first clinical study using CTP to investigate the acute effects of high-dose irradiation using SABR in RPC and BRPC patients and measure their vascular response. Notably, the post-1^st^-fx BF was found to be significantly higher relative to the pre-treatment baseline in Groups 1 and 2, and relative to post-cx in Group 3. On the other hand, the PS showed a significant decrease at post-1^st^-fx relative to post-cx in all BRPC patients (Group 3). These observations demonstrate that just a single fraction of neoadjuvant SABR can induce an acute change in pancreatic tumor perfusion. However, the trends observed at 3–4 weeks post-RT in Group 3—namely the overall non-significant decrease in tumor BF and the inconsistent changes in PS among patients—suggest that the initial post-irradiation response may only be transient.

For both RPC and BRPC patients, the observed vascular responses are likely a result of the treatment effect on the desmoplastic stroma. For instance, the overall increase in tumor perfusion that was observed at post-cx relative to baseline in Group 2 may at least be partly explained by vascular normalization—a prominent treatment strategy for solid tumors that aims to restore vessel integrity by reverting aberrant tumor vessels to their normal, functioning state (32–34). However, as to why Patients 1 and 2 did not seem to show a further increase in tumor BF at post-1^st^-fx relative to post-cx, there may be several explanations. First, it is possible that there was a decrease in BF over time following chemotherapy. In this case, the post-1^st^-fx increase in tumor BF may not have been adequately captured due to it being comparable to what was initially induced by the chemotherapy. Thus, acquiring a CTP scan immediately prior to irradiation could help circumvent such an issue in future studies. Another likely explanation is that Patients 1 and 2 might have had an exceptionally positive response to chemotherapy. In other words, the chemo drugs might have significantly normalized the desmoplastic stroma to such an extent that no acute vascular response could be observed at only 6 hours after delivering the first fraction of SABR. Since the RPC patient, who only received neoadjuvant SABR in this study, did demonstrate a post-1^st^-fx increase in tumor BF relative to the pre-treatment baseline; a more prominent post-1^st^-fx response may possibly be observed if the order of treatment was reversed for the BRPC patients in a future study, with SABR preceding chemotherapy. To simplify the clinical workflow in implementing this change, the standard-of-care multiphase contrast-enhanced CT protocol could be modified to incorporate CTP without requiring patients to undergo additional scans for measuring tumor perfusion.

To discuss the differences in response between Patient 1 and Patient 2 specifically, it is important to note that the total prescription dose for Patient 1 had to be decreased to about 25 Gy due to the high duodenal dose. Although both Patients 1 and 2 showed a similar level of acute response at post-1^st^-fx, the reason why Patient 2 seemed to demonstrate a further increase in tumor BF, BV and PS at post-RT—which were not observed in Patient 1—may be attributed to the fact that they received a higher total dose of 33 Gy. Like Patient 2, the RPC patient and BRPC Patients 3–5 also received comparably high dose prescriptions (≥ 30 Gy) but showed a more prominent post-1^st^-fx increase in BF compared to Patient 2. While these observations are still anecdotal, they suggest that the total dose prescription may have an impact on the overall tumor response. In the case of Patient 2, the lack of a noticeable post-1^st^-fx response may be due to patient-specific variability, the cause of which may be better characterized by investigating a larger sample of patients in a future study.

Additionally, determining the precise duration and magnitude of the SABR-induced change in tumor perfusion will require further investigation as the acute vascular responses to high-dose, single-fraction irradiation are still underexplored in the literature. As of now, findings from a limited number of studies suggest that an interplay of various biological processes may underlie the post-SABR increase in tumor perfusion. For instance, the induction of apoptosis in tumor endothelial cells (TECs), peaking at approximately 4–6 hours after single-dose irradiation at 15 Gy, has been shown to be mediated by radiation-induced alterations in the cell membrane as opposed to DNA damage (35, 36). At such high doses, TECs of large-diameter vessels enter cell cycle arrest but remain perfused; whereas, the hypo-perfused vessels of smaller diameter are pruned away due to their greater susceptibility to apoptosis (37). In effect, this can lead to an increase in tumor perfusion as blood flow becomes re-routed to the larger vessels that remain intact post-irradiation (37). Thus, the re-routing of BF to the larger vessels may explain why Groups 1 and 3 consistently showed a significantly higher BF, but not BV, at 6 hrs post-1^st^-fx. Additionally, while radiation-induced damage to the endothelium is associated with an increase in vascular permeability (38), vessel pruning may have led to an even greater reduction in the total surface area of perfused vessels; thus, contributing to an overall decrease in PS, which was observed at post-1^st^-fx in Group 3.

Although the observed increase in BF and decrease in PS at post-1^st^-fx oppose each other in terms of their impact on the overall tumor perfusion, the net effect of these changes can be described by the V_e_ in terms of systemic delivery of chemotherapeutic drugs. A more vigorous discussion of the relationship between BF, PS and V_e_ can be found in the **Supplementary Material**. While the observed changes in V_e_ at post-1^st^-fx were found to be non-significant, our study demonstrated that the V_e_ at 3–4 weeks post-RT was indeed significantly higher relative to the pre-treatment baseline in Group 1, and relative to post-cx in Group 3. As outlined in the **Supplementary Material**, under distribution equilibrium—such as in systemic chemotherapy delivery—V_e_ acts as a partition coefficient between blood and tissue. Thus, a higher V_e_ reflects greater drug partitioning into tissue or improved systemic drug delivery to the tumor cells. Physiologically, an increase in V_e_ indicates that the extravasated contrast agent is distributed across a larger interstitial (extravascular extracellular) volume. This volume is anatomical, with an upper limit of 1.0 mL/g (assuming a tissue density of 1.0 g/mL), and represents the complement of cell density (CD). Thus, an increase in V_e_ can be interpreted as a decrease in the tumor CD—which is characteristically high in PDAC due to desmoplasia. Interestingly, the largest drops in CD were observed after patients received their primary treatment in Group 1, namely post-RT for the RPC patient, and post-cx for BRPC Patients 1 and 2. Smaller drops in CD relative to post-cx could also be observed for the BRPC patients after they completed SABR, their secondary treatment. In terms of V_e_, this post-RT change relative to post-cx was found to be statistically significant in Group 3. Since it is expected that the primary treatment would lead to a greater initial decrease in tumor volume that can be detected by imaging compared to the secondary treatment, the observed trends in CD show that CTP may have adequate sensitivity as an imaging modality to provide surrogate measures of PDAC tissue density as CD. However, the inconsistent changes observed at 6 hrs post-1^st^-fx suggest that CD may have greater accuracy in monitoring patient response over longer timescales, in the order of days to weeks, as would be required for macroscopic changes to occur in the tumor volume and be detected by CTP. The observed changes in cellular density must also be validated against histology-measured ground truth (e.g., from biopsies or excised tumors) to assess their reliability as a biomarker.

Additionally, as a biomarker, CD represents the ability of the delivered radiation to ablate the tumor cells, which may directly lead to changes in the IFP. Although irradiation involving low doses was not explored in this study, there is evidence in the literature to suggest that radiation-induced changes in tumor perfusion are dose-dependent. For instance, a study by Znati et al. (29) showed that there was a reduction of IFP following single-fraction irradiation with doses greater than 10 Gy. In a separate experiment using doses lower than 10 Gy, however, no change in the IFP could be observed (29). The study attributed the decrease in IFP to a reduction in the compression of the venous vessels by the tumor cells and noted that the decrease in IFP preceded any observable macroscopic changes in the tumor volume (29), implying the acute nature of the decrease in IFP following high-dose irradiation. This suggests that while the significant increases in V_e_ were only observed 3–4 weeks after completing the full 3-fx SABR in our study; the acute vascular changes that occurred at 6 hours post-1^st^-fx, including the increase in BF and decrease in PS, may then lead to other important changes in pancreatic tumor perfusion, such as a decrease in IFP—which could enhance the convective transport of systemic chemo drugs to the tumor site (23). Nevertheless, the exact timeframe and duration of the observed vascular responses will have to be further investigated.

In summary, our current analysis suggests that 3-fx neoadjuvant SABR could feasibly improve pancreatic tumor perfusion, with radiation-induced vascular changes occurring as acute as 6 hours after delivering the first fraction of SABR. While these findings have yet to be validated with a larger sample size of patients, this initial study has demonstrated the possibility of utilizing CTP as a means of monitoring patient response to standard-of-care treatment for RPC and BRPC. Our study also provides preliminary evidence that supports the possibility of delivering neoadjuvant SABR to induce an acute increase in pancreatic tumor perfusion prior to administering systemic NAT. Considering the acute nature of the SABR-induced vascular effect—as demonstrated by our study and several others in the literature—this may involve systemic NAT being delivered on the same day that the patient receives SABR for an optimal synergistic effect. Nevertheless, these hypothesis-generating findings warrant larger clinical studies to further test and validate the SABR-first sequencing. Additionally, future studies should include pharmacokinetic and histopathological correlation of the observed perfusion changes by incorporating measurements such as gemcitabine tumor-to-plasma ratios or nab-paclitaxel immunohistochemistry.

In terms of discussing the validity of the calculated CTP parameters, a test-retest reproducibility study involving two scans in the same session has yet to be performed in the current literature, as it would subject patients to additional contrast and radiation without clear benefit, raising ethical concerns. However, studies in the literature have assessed the reproducibility of pancreatic CT perfusion (CTP) parameters, both when the same dataset was analyzed by the same observer across different sessions and by different observers. For instance, Garcia et al. reported that for blood flow measurements, the intraobserver difference was approximately 1 mL/min/100g, while the interobserver 95% limit of agreement was considerably broader, around 56 mL/min/100g (39). Importantly, because all CTP analyses in our study were performed by a single observer, the substantial interobserver variability observed in previous studies did not influence our findings.

Furthermore, while the original proposal for this study was to deliver the 3-fx neoadjuvant SABR prior to chemotherapy, the treatment regimen was mandated to administer chemotherapy first, prior to neoadjuvant SABR, due to a lack of clinical evidence showing potential benefit to patient outcome. Current NCCN guidelines also favor total neoadjuvant therapy with chemotherapy first for BRPC patients, and the SABR-first regimen is currently only reasonable in clinical trials (40). Therefore, establishing the foundation to deliver neoadjuvant SABR prior to chemotherapy in PDAC patients will require future prospective studies to investigate patient outcome measures, such as disease-free survival (DFS) and pathological complete response (pCR) post-surgery. These measures will also help assess the safety of administering neoadjuvant SABR to pancreatic cancer patients as increasing tumor perfusion may also be associated with potential metastases and cancer recurrence (41, 42). To address this risk, even though vascular responses have shown to be induced by a single fraction of high-dose irradiation, a multi-fraction radiotherapy plan may still be required to treat potential micro-metastases that could result after the initial single-dose irradiation. Delivering systemic NAT, such as chemotherapy, shortly after the irradiation would also help account for these micro-metastases.

Finally, in addition to boosting tumor perfusion to potentially improve patient response to systemic NAT, delivering neoadjuvant SABR prior to chemotherapy offers several treatment benefits for the upfront RPC and BRPC patients. These include: i) providing radiotherapy upfront for patients who may be found to have post-surgery complications that render them ineligible for adjuvant therapy, ii) avoiding unnecessary surgical intervention if metastases can be detected during post-therapy restaging, and iii) effectively downstaging the tumor conditions for increased chances of achieving an R0 resection. In conclusion, with our study providing preliminary evidence that neoadjuvant SABR can induce an acute increase in pancreatic tumor perfusion, administering neoadjuvant SABR prior to systemic therapy, such as chemotherapy or immunotherapy, may help overcome PDAC chemoresistance due to desmoplasia. Ultimately, this would require larger studies to validate these initial exploratory analyses and demonstrate that the SABR-first sequencing could lead to improved rates of R0 resection and better overall survival for patients with RPC and BRPC.

## Data Availability

The original contributions presented in the study are included in the article/**Supplementary Material**. Further inquiries can be directed to the corresponding author.
