# Supplementary material for: Analyzing the effect of neoadjuvant stereotactic ablative body radiotherapy on pancreatic tumor perfusion using computed tomography perfusion
Source: Front Oncol. 2026 Mar 12;16:1677923. doi: 10.3389/fonc.2026.1677923 (PMC13017369; doi:10.3389/fonc.2026.1677923)
Supplement: Supplementary file 1 [file DataSheet1.docx]

**A.0 Supplementary Material**

**A.1 Derivation of Distribution Volumes in CTP**

In the following, we show how blood volume (BV) and the extravascular extracellular volume (V_e_) are related to the area under the curve of $R_{F}(t)$, or the flow-scaled impulse residue function that is output from the deconvolution algorithm. While the derivation is necessarily brief, refer to the reference by Lee et al^31^ for more information. First, $R_{F}(t)$ can be expressed as:

$$R_{F}\left( t \right)=FH\left( t-t_{0} \right)+FH\left( t-W-t_{0} \right)+FE*e^{-k_{2}\left( t-W-t_{0} \right)}$$

where $F$ is blood flow, $E$ is the extraction efficiency of the contrast agent by tissue, $k_{2}$ is the efflux rate constant of contrast from tissue back to blood, $W$ is the mean transit time, $t_{0}$ describes the delay in the arrival of the contrast at the tissue after injection relative to the artery (aorta in this case), and $H(t)$ denotes the Heaviside function. By definition, the total contrast distribution volume ($V_{D}$) in tissue for x-ray contrast that remains extracellular is given by:

$$V_{D}=BV+V_{e}$$

Lee et al^31^ also shows that:

$$V_{D}=\int_{0}^{\infty} R_{F}(t) dt$$

$$=\int_{0}^{\infty} FH\left( t-t_{0} \right)+FH\left( t-W-t_{0} \right)+FE*e^{-k_{2}\left( t-W-t_{0} \right)} dt$$

$$=\int_{0}^{\infty} FH\left( t-t_{0} \right)+FH\left( t-W-t_{0} \right) dt+\int_{0}^{\infty} FE*e^{-k_{2}\left( t-W-t_{0} \right)} dt$$

$$=\int_{t_{0}}^{t_{0}+W} FH\left( t-t_{0} \right)+FH\left( t-W-t_{0} \right) dt+FE\int_{t_{0}+W}^{\infty} e^{-k_{2}\left( t-W-t_{0} \right)} dt$$

$$=FW+FE\left( -\frac{1}{k_{2}}e^{-k_{2}\left( t-W-t_{0} \right)} \right)\text{|}\begin{matrix} \infty\\ \\ t_{0}+W \end{matrix}$$

$$=FW+FE\left( 0-\left( -\frac{1}{k_{2}} \right) \right)$$

$$V_{D}=FW+\frac{FE}{k_{2}}=BV+\frac{FE}{k_{2}}$$

Note that by the Central Volume Principle, the mean transit time ($W$) is given by:

$$W=\frac{BV}{F}$$

Therefore, the extravascular extracellular volume ($V_{e}$) is given by:

$$V_{e}=\frac{FE}{k_{2}}$$

Because the default output for the distribution volumes are in units of mL/100 g of tissue, normalizing $V_{e}$ to unit tissue mass in grams (division by 100) and subtracting it from 1 mL/g—or the volume that would be taken up by tissue per gram if it was composed entirely of water—would theoretically yield the cell density, or the volume taken up by the cells per g of tissue (also known as the intracellular volume):

$$Cell Density (in units of\frac{mL}{g}of tissue)\approx1-\frac{V_{e}}{100}$$

**A.2 Extravascular Extracellular Volume: Marker for Net Contrast Delivery to Tissue**

In the following, we show that an increase in the extravascular extracellular volume ($V_{e}$) is equivalent to a net increase in contrast delivery to the interstitial space. By mass balance and Fick’s law of diffusion (for details refer to Lee et al^31^),

$$\frac{d\left( C_{e}\left( t \right) \right)}{dt}=K_{1}C_{a}\left( t \right)-k_{2}C_{e}\left( t \right)$$

where $K_{1}=FE$


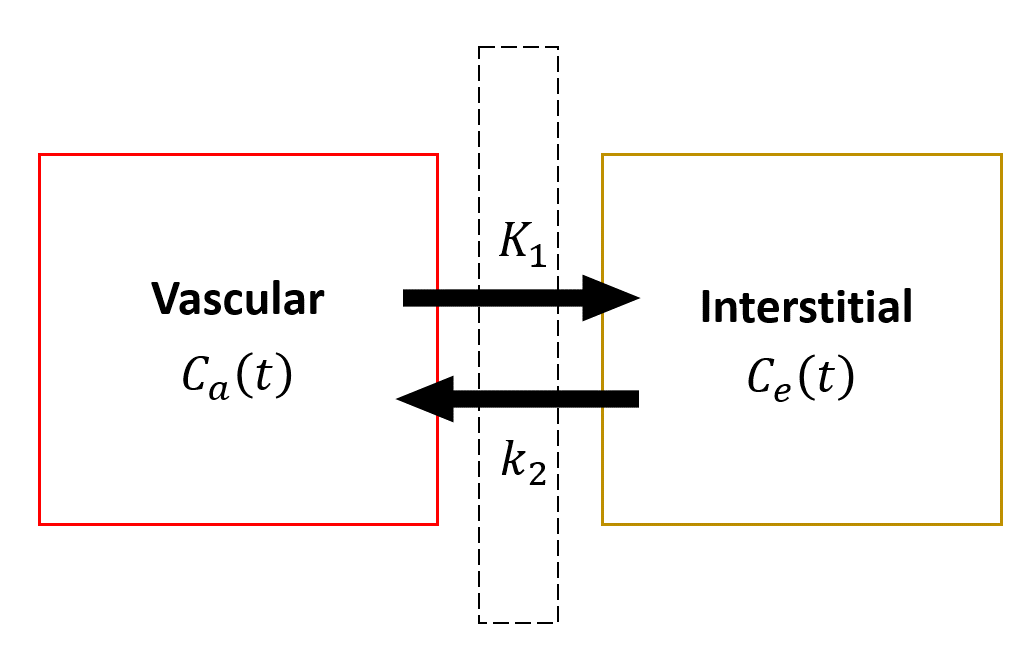


In the context of systemic chemo-drug delivery, a system is in dynamic equilibrium:

$$\frac{d\left( C_{e}\left( t \right) \right)}{dt}=K_{1}C_{a}\left( t \right)-k_{2}\left( C_{e}\left( t \right) \right)=0$$

$$K_{1}C_{a}\left( t \right)=k_{2}\left( C_{e}\left( t \right) \right)$$

$$\frac{C_{e}\left( t \right)}{C_{a}\left( t \right)}=\frac{K_{1}}{k_{2}}=\frac{FE}{k_{2}}$$

$$\frac{C_{e}\left( t \right)}{C_{a}\left( t \right)}=V_{e}$$

Therefore, this means that an increase in $V_{e}$ is indicative of an increase in the contrast concentration within the interstitial space given that $C_{a}\left( t \right)$ remains the same. Additionally, given that $K_{1}=FE$, where $E$ is a function of $F$ and the permeability-surface area product ($PS$); the $V_{e}$ also helps account for the net effect that changes in $F$ and $PS$ would have on systemic drug delivery.

$$V_{e}=\frac{K_{1}}{k_{2}}=\frac{FE}{k_{2}}=\frac{F\left( 1-e^{-\frac{PS}{F}} \right)}{k_{2}}$$
