# Supplementary material for: Analyzing the effect of neoadjuvant stereotactic ablative body radiotherapy on pancreatic tumor perfusion using computed tomography perfusion
Source: Front Oncol. 2026 Mar 12;16:1677923. doi: 10.3389/fonc.2026.1677923 (PMC13017369; doi:10.3389/fonc.2026.1677923)
Supplement: Supplementary file 2 [file DataSheet2.docx]

**Table 2.** Individual patient demographic and clinical outcome data

| **Patient** | Age | Chemotherapy Regimen | Number of Chemo Cycles | Cancer Stage (AJCC) | Tumor Grade at Surgery | Residual Tumor Classification |
| --- | --- | --- | --- | --- | --- | --- |
| BRPC Patient 1 | 69 | FFX | 3 | cT2N0M0 | Grade 2 | R0 |
| BRPC Patient 2 | 73 | G/A | 4 | cT2N0M0 | Grade 3 | R0 |
| BRPC Patient 3 | 73 | G/A | 6 | cT1N0M0 | Grade 2 | R0 |
| BRPC Patient 4 | 60 | FFX | 12 | cT4N0M0 | Grade 2 | R0 |
| BRPC Patient 5 | 70 | FFX | 8 | cT4N0M0 | Grade 1 | R0 |
| RPC Patient | 69 | - | - | pT2N1cM0 | Grade 2 | R0 |

**Table 3.** CTP parameters calculated at each study timepoint for individual patients

| **BRPC Patient 1** | Baseline | Post-Chemo | Post-1^st^-fx | Post-RT |
| --- | --- | --- | --- | --- |
| BF (mL/min/100g) | 18.61 | 56.00 | 57.15 | 40.97 |
| BV (mL/100g) | 2.53 | 8.81 | 7.24 | 6.07 |
| PS (mL/min/100g) | 5.71 | 21.53 | 16.14 | 14.77 |
| V_e_ (mL/100g) | 9.90 | 34.33 | 27.79 | 36.75 |
| CD (mL/g) | 0.90 | 0.66 | 0.72 | 0.63 |
| **BRPC Patient 2** | Baseline | Post-Chemo | Post-1^st^-fx | Post-RT |
| BF (mL/min/100g) | 24.72 | 53.98 | 54.76 | 63.31 |
| BV (mL/100g) | 2.98 | 10.83 | 9.24 | 9.63 |
| PS (mL/min/100g) | 7.45 | 25.29 | 21.16 | 26.90 |
| V_e_ (mL/100g) | 6.82 | 23.63 | 23.74 | 25.81 |
| CD (mL/g) | 0.93 | 0.76 | 0.76 | 0.74 |
| **BRPC Patient 3** | Baseline | Post-Chemo | Post-1^st^-fx | Post-RT |
| BF (mL/min/100g) | - | 34.11 | 63.89 | - |
| BV (mL/100g) | - | 5.68 | 11.87 | - |
| PS (mL/min/100g) | - | 17.54 | 15.11 | - |
| V_e_ (mL/100g) | - | 16.60 | 24.26 | - |
| CD (mL/g) | - | 0.83 | 0.76 | - |
| **BRPC Patient 4** | Baseline | Post-Chemo | Post-1^st^-fx | Post-RT |
| BF (mL/min/100g) | - | 42.03 | 78.07 | 44.37 |
| BV (mL/100g) | - | 9.79 | 10.72 | 7.91 |
| PS (mL/min/100g) | - | 31.35 | 27.05 | 29.16 |
| V_e_ (mL/100g) | - | 33.32 | 39.70 | 39.65 |
| CD (mL/g) | - | 0.67 | 0.60 | 0.60 |
| **BRPC Patient 5** | Baseline | Post-Chemo | Post-1^st^-fx | Post-RT |
| BF (mL/min/100g) | - | 53.98 | 94.28 | 88.11 |
| BV (mL/100g) | - | 8.94 | 14.59 | 9.75 |
| PS (mL/min/100g) | - | 34.44 | 29.26 | 26.79 |
| V_e_ (mL/100g) | - | 40.88 | 37.77 | 44.62 |
| CD (mL/g) | - | 0.59 | 0.62 | 0.55 |
| **RPC Patient** | Baseline | Post-Chemo | Post-1^st^-fx | Post-RT |
| BF (mL/min/100g) | 74.89 | - | 98.15 | 96.24 |
| BV (mL/100g) | 19.36 | - | 18.04 | 20.13 |
| PS (mL/min/100g) | 15.01 | - | 15.04 | 26.29 |
| V_e_ | 20.98 | - | 17.12 | 31.87 |
| CD | 0.79 | - | 0.83 | 0.68 |
